# Re-evaluation of the occupational exposure limit for ZnO is warranted. Comments on 'Systemic inflammatory effects of zinc oxide particles: is a re-evaluation of exposure limits needed?' by Christian Monsé et al.

**DOI:** 10.1007/s00204-023-03634-w

**Published:** 2023-12-01

**Authors:** Ulla Vogel, Anne T. Saber, Nicklas R. Jacobsen, Pernille H. Danielsen, Karin S. Hougaard, Niels Hadrup

**Affiliations:** National Research Centre for the Working Environment, Copenhagen, Denmark

Christian Monsé and co-workers recently published a Short Communication in Archives of Toxciology (Monse et al. 2023) commenting on the suggested health-based occupational exposure limit for zinc oxide made by the National Research Centre for the Working Environment (NFA) in Denmark (Hadrup et al. 2021b).

In 2018, Christian Monsé and co-workers made a controlled human exposure study (Monse et al. 2018), which reproduced the dose-dependent ZnO-induced acute phase response in human volunteers that we have previously observed in mice (Hadrup et al. 2019; Jacobsen et al. 2015; Saber et al. 2022), and enabled NFA to derive a health-based occupational exposure limit based on human data. Our suggestion was 0.05 mg/m^3^ ZnO. In the recent short communication, Monsé and et al. argues that a higher OEL for ZnO could be justified. We would like to address some of their arguments.

We agree with Monsé et al. that the No Observed Effect Concentration (NOEC) for systemic acute phase response is 0.5 mg/m^3^ ZnO for 4 h. We furthermore consider it a No Observed Adverse Effect Concentration (NOAEC), because we consider the induced effects as adverse. As also mentioned by Monsé et al., Brand et al. showed, in their controlled human exposure studies, that the biologically relevant dose is the total daily dose calculated as time x concentration (Brand et al. 2019). We argue that the same dose–response relationship would be expected in the range of the NOAEC, and therefore, the derived NOAEC for an 8-h working day should be 0.25 mg/m^3^ (ECHA 2012).

ZnO exposure induces dose-dependent acute phase response in humans and mice (Hadrup et al. 2019; Jacobsen et al. 2015; Saber et al. 2022; Monse et al. 2023). The acute phase response is the systemic response to acute and chronic inflammatory states caused by, e.g. bacterial infection, trauma and infarction (Gabay and Kushner 1999). The acute phase protein Serum Amyloid A (SAA) is causally related to atherosclerosis and cardiovascular disease (Hadrup et al. 2020; Thompson et al. 2018). Overexpression of SAA promotes atherosclerosis in mouse models (Christophersen et al. 2021; Thompson et al. 2018). Blood levels of SAA and C-reactive protein (CRP) are closely correlated in blood. In prospective, epidemiological studies, even very moderate increases in SAA and CRP concentrations in blood are associated with increased risk of cardiovascular (Pai et al. 2004; Ridker et al. 2000). Since cardiovascular diseases such as atherosclerosis, apoplexia and ischemic heart disease are extremely prevalent, even small increases in the risk of cardiovascular disease are relevant, also in the working environment.

Monsé et al. argue that the ZnO-induced acute phase response is a substance-specific effect. We disagree. We acknowledge that a particle-dependent acute phase response differs for soluble and insoluble materials, e.g. as shown for metal oxides (Gutierrez et al. 2023). Soluble metal oxides, such as CuO or ZnO, that undergo dissolution in lysosomes induce a strong acute phase response in humans and mice (Brand et al. 2019; Gutierrez et al. 2023; Hadrup et al. 2021a). The acute phase response of soluble particles is transient and independent of particle size (Monse et al. 2021). On the other hand, in mice, insoluble particles will induce long-lasting acute phase response, which is predicted by the total surface area of the deposited particles (Danielsen et al. 2019; Gutierrez et al. 2023; Hadrup et al. 2021a; Poulsen et al. 2017; Saber et al. 2014). There is also evidence in humans for particle-induced acute phase response by insoluble particles. In a controlled human study, 4 h of exposure to 38 μg /m^3^ PM2.5 caused a 9% increase in SAA levels (Wyatt et al. 2020). In a study of air pollution and CRP levels in 30,000 Taiwanese, a 5 μg increase in PM2.5 levels was associated with 1.3% increased CRP levels (Zhang et al. 2017).

Monsé et al. argue that ‘the transferal from young and healthy subjects to the general working population should not represent a relevant problem’ and argues against the use of assessment factor to account for inter-individual variation. We would like to point out that lifestyle factors such as BMI, smoking and exercise modify the acute phase response (Jylhava et al. 2009) and that low-grade inflammation, which includes slightly elevated acute phase response, is a risk factor for other major diseases such as type 2 diabetes. We have little knowledge on possible combination effects. We also note that a factor of 5 is the default intra-species assessment factor for workers by ECHA (ECHA 2012, 2019).

In conclusion, Monsé and co-workers have performed important controlled studies on ZnO that replicated the ZnO-induced acute phase response in humans that we have observed in mice. Compared to the current Danish occupational exposure limit for ZnO at 5 mg/m^3^, an exposure limit for ZnO of 0.1 mg/m^3^ as suggested by the MAK commission (Monse et al. 2023) and a health-based occupational exposure limit at 0.05 as suggested by us (Hadrup et al. 2021b) may be regarded as being very similar.
